# Finite Element Analysis of Thermal–Mechanical Coupling and Process Parameter Optimization in Laser Etching of Al–Tedlar–Kevlar Composite Films

**DOI:** 10.3390/ma18214839

**Published:** 2025-10-23

**Authors:** Ming Liu, Rui Wang, Shanglin Hou, Kaiwen Shang, Dunzhu Gesang, Guang Wei

**Affiliations:** 1 Science and Technology in Vacuum Technology and Physics Laboratory, Lanzhou Institute of Physics, Lanzhou 730000, China; wriop@126.com (R.W.); shangkaiwen@126.com (K.S.); xd_wg@163.com (G.W.); 2 Science and Technology on Surface Engineering Laboratory, Lanzhou Institute of Physics, Lanzhou 730000, China; 3 School of Microelectronics Industry-Education Integration, Lanzhou University of Technology, Lanzhou 730000, China; housl@lut.edu.cn

**Keywords:** laser processing, composite materials, thermal–mechanical coupling

## Abstract

Laser processing of heterogeneous composites requires a clear understanding of coupled thermal and mechanical responses to ensure structural integrity and patterning precision. In this study, a thermal–mechanical coupling model based on the finite element method was developed to investigate laser–material interactions in Al–Tedlar–Kevlar composite films. The effects of key parameters—including pulse energy, spot size, pulse duration, and repetition frequency—on the evolution of temperature and stress fields were systematically examined. The simulations reveal that pulse energy leads to a linear rise in peak temperature, while pulse duration exerts a nonlinear influence on energy density and thermal uniformity. Increasing repetition frequency promotes thermal accumulation, enlarging the heat-affected zone. Coupled analyses further indicate significant stress concentrations at material interfaces, which may trigger delamination and compromise film reliability. Through comprehensive parameter evaluation, the optimal processing conditions were identified as 0.5 mJ pulse energy, 20 kHz repetition rate, 45 μm spot diameter, and 120 ns pulse duration. These findings clarify the governing mechanisms of thermal–mechanical interactions in multilayer composites and provide theoretical guidance for optimizing laser micropatterning processes while enhancing interfacial stability and manufacturing quality.

## 1. Introduction

The demand for lightweight, high-strength, and high-temperature-resistant materials has been continuously increasing in fields including flexible electronics, aerospace, and intelligent multifunctional packaging [1,2]. Polymer/metal multilayer composite films have gradually emerged as important material systems in advanced manufacturing due to their excellent mechanical properties, thermal stability, and electromagnetic shielding capabilities [3,4,5]. Among these, the Al–Tedlar–Kevlar heterogeneous composite film combines the advantages of three materials: aluminum exhibits exceptional thermal conductivity, the Tedlar film offers both flexibility and chemical inertness, while Kevlar fibers possess a high elastic modulus and excellent impact resistance. Consequently, this composite system exhibits significant application potential in optoelectronic packaging, electromagnetic shielding, and thermal management for microsystems [6].

Laser etching technology, recognized for its efficiency and precision, has been widely applied in the surface treatment and microstructure fabrication of various materials [7,8,9]. However, significant differences in physical properties, such as thermal conductivity, thermal expansion coefficient, Young’s modulus, and thermal stability among the layers of Al–Tedlar–Kevlar composite films can easily lead to thermal stress concentration, local warping, and even interface delamination during the laser processing. These failures significantly affect the structural stability and service life of microstructures [10,11]. Therefore, a comprehensive understanding of the thermo-mechanical coupling mechanisms during the laser-material interaction process is of significant importance for improving processing accuracy, controlling the heat-affected zone, and optimizing process parameters.

In recent years, numerous scholars have conducted in-depth modeling and numerical simulations of laser-induced heating, ablation, and phase transition processes [12,13]. Bian et al. [14] employed laser-induced spallation experiments combined with modeling to investigate the evolution of interfacial stresses and delamination mechanisms in polymer–metal multilayer films under pulsed laser irradiation. Chakraborty et al. [15] developed a thermal conduction model to analyze heat transfer and potential substrate damage during laser-assisted copper patterning on flexible polymer substrates. Recent studies combining finite element simulations with experiments have also advanced rapidly. For instance, Li et al. [16] integrated three-dimensional simulations and laser ablation experiments to reveal the dynamic evolution of temperature fields and degradation behavior in polymer microchannel fabrication. Moreover, the finite element method has been widely applied to the laser processing of thin-film materials, including the laser crystallization of silicon films [17], the pulsed laser etching of metal/polyimide composites [18], the simulation of photothermal synthesis in metal–organic framework films [19], and the numerical modeling of interfacial delamination in functional multilayers under femtosecond laser irradiation [20]. However, there remains a lack of systematic studies on the effects of laser parameters on the temperature field evolution, stress distribution characteristics, and interface stability of heterogeneous composite films, particularly for the Al–Tedlar–Kevlar system.

Based on this, the present work investigates Al–Tedlar–Kevlar heterogeneous composite films by establishing a multiphysics coupling model using the finite element method. The effects of key laser parameters—including pulse energy, pulse duration, and repetition frequency—on the regulation mechanisms of temperature and stress fields are systematically examined. The study elucidates how laser parameters influence structural stability and etching quality of the multilayer films, thereby providing theoretical support and process optimization guidance for high-precision laser machining of heterogeneous composite films.

## 2. Model and Methods

### 2.1. Physical Model and Geometric Modeling

**Figure 1:**
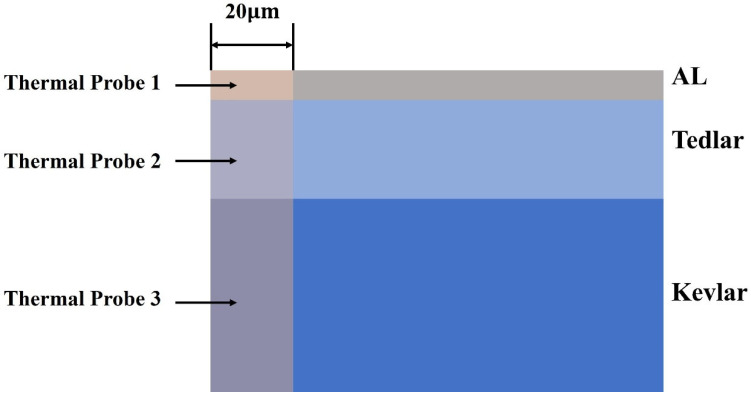
Composite material system model.

The model consists of a three-layer structure, as illustrated in Figure 1, with the laser incident vertically onto the Al surface. The thicknesses of each layer are as follows: aluminum film: 2 μm; Tedlar: 25 μm; Kevlar: 100 μm. This configuration enhances computational efficiency while ensuring accuracy.

Prior to the pulsed laser heating, the temperatures of aluminum, Tedlar material, and the surrounding environment are at room temperature, with an initial temperature defined as:

(1) $$T_{\left(x,y,z\right)}{|}_{t=0}=293K$$

The heat flow and temperature at the interface between the Al film and the Tedlar substrate exhibit continuous distribution, expressed as [21]

(2) $$n\cdot \left(k_{AL}\cdot \nabla T\right)=n\cdot \left(k_{Ted}\cdot \nabla T\right)=n\cdot \left(k_{Kev}\cdot \nabla T\right)$$

where n is the unit normal vector at the boundary, and $k_{AL}$, $k_{Ted}$, and $k_{Kev}$ represent the thermal conductivities of Al, Tedlar, and Kevlar, respectively.

### 2.2. Material Properties and Laser Heat Source Configuration

The physical parameters of the three materials are listed in Table 1 [22,23]. It should be noted that in our calculation, we have taken into account the temperature-dependent characteristics of Al and Kevlar material properties.

The laser wavelength is 1064 nm, with a pulse duration of 120 ns, pulse energy of 1 mJ, and a repetition frequency of 20 kHz (with a period of 50 μs). The scanning speed is 500 mm/s [24].

The laser spot is described by a Gaussian distribution [25], which is given by:

(3) $$f\left(x\right)=\frac{1}{2\pi \sigma^{2}}exp\left(-\frac{x^{2}}{2\sigma^{2}}\right)$$

Here, σ represents the standard deviation of the Gaussian pulse, with a full width at half maximum (FWHM) given by FWHM = $2\sqrt{2ln2\sigma }$. The laser spot size ranges from 40 μm to 50 μm, and calculations are conducted for three specific diameters: 40 μm, 45 μm, and 50 μm. The relationship between the laser spot size and standard deviation is as follows:

σ = 16.986 μm, FWHM = 2.3548 × 16.986 = 40 μm
σ = 19.11 μm, FWHM = 2.3548 × 16.986 = 45 μm
σ = 21.233 μm, FWHM = 2.3548 × 21.233 = 50 μm

The laser input was modeled using a Gaussian heat source, which is a well-established and computationally efficient approximation in laser–material interaction studies. Although actual beam profiles and absorption characteristics are influenced by factors such as incidence angle, surface roughness, and phase transitions, this assumption provides a sufficiently accurate first-order representation to capture the essential thermo–mechanical coupling mechanisms addressed in this work.

### 2.3. Multi-Physics Coupling and Boundary Conditions

**Figure 2:**
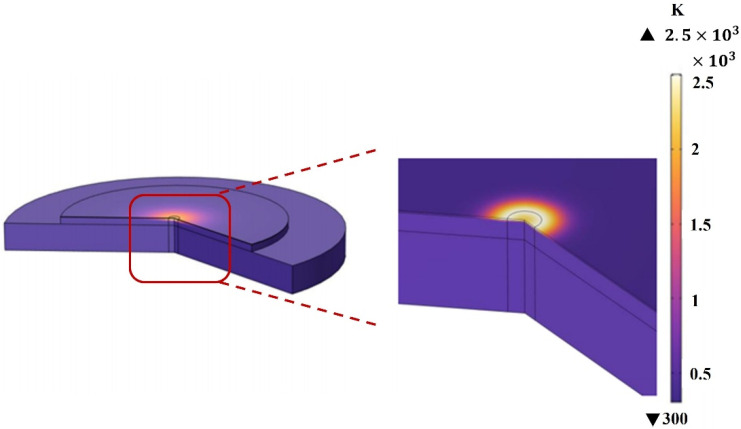
Temperature distribution in the heterogeneous film.

A multiphysics coupling model was developed using the finite element method [26,27], and numerical simulations were performed. The laser beam is incident perpendicularly along the -z axis onto the Al surface of a three-layer film, with a 2D axisymmetric model. The temperature distribution is solved by combining heat conduction and the Beer-Lambert law, considering infrared reradiation and pulsed heat sources at the surface interface. The films are assumed semi-transparent, with reflection at the interface and no refraction or diffraction. The coherence length is smaller than the layer thickness. The simulation parameters include an incident laser wavelength of 1064 nm, pulse width of 120 ns, pulse energy of 1 mJ, and repetition frequency of 20 kHz. The mesh resolution is 0.8 μm for the polymer layers (Tedlar and Kevlar) and 0.2 μm for aluminum. The laser spot size is 45 μm. The resulting temperature distribution is shown in Figure 2.

### 2.4. Simulation Strategy

**Figure 3:**
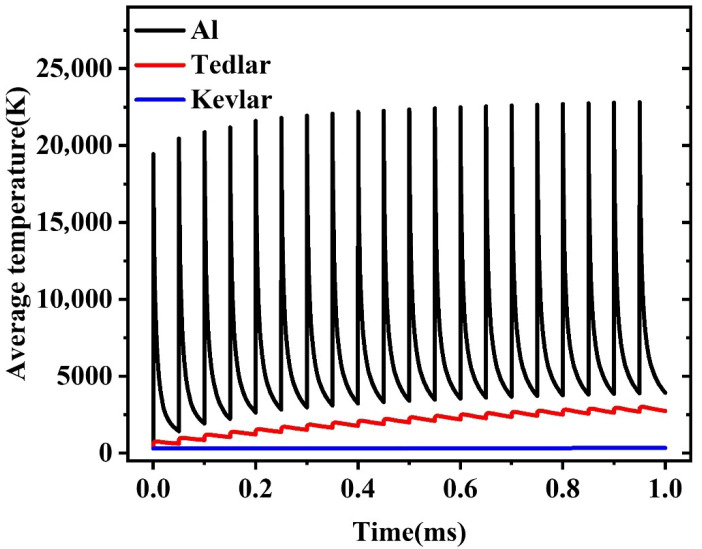
Average temperatures of Al, Tedlar, and Kevlar at the aluminum film spot under 1064 nm laser pulses (pulse energy: 1 mJ, pulse width: 120 ns, laser spot: 45 μm, repetition rate: 20 kHz).

A spot size area of 45 μm is selected, and a probe, as shown in Figure 1, is introduced to analyze the average temperature within the area where the spot is located in the three-layer structure. The temperature variation in the spot area over the time period of 0 to 1 ms is illustrated in Figure 3.

Since the scanning frequency is 500 mm/s, the dwell time of the laser at a single spot (45 μm area) is given by:

(4) $$\frac{45\;\mu m}{500\;mm/s}=90\;\mu s$$

This means that the spot remains for no more than 2 laser pulse periods, or 100 μs. Subsequent analyses will focus on the temperature within the 100 μs duration corresponding to these two laser pulse cycles.

The coupled solid mechanics model was employed to perform stress analysis. The physical parameters for Tedlar are as follows: Young’s modulus of 3.6 GPa, Poisson’s ratio μ = 0.38, and thermal expansion coefficient α = 7 × 10^−5^ 1/K. The physical parameters for Al and Kevlar were obtained from standard material databases.

## 3. Results and Discussion

### 3.1. Impact of Laser Pulse Energy on Al–Tedlar–Kevlar Composite Film

To systematically investigate the effect of laser pulse energy on the thermal response of the Al–Tedlar–Kevlar composite film, finite element numerical simulations were conducted under the conditions of a laser frequency of 20 kHz and pulse width of 120 ns. The analysis focused on the temperature field distribution in each layer and the evolution of the heat-affected zone as the pulse energy varied within the range of 0.1–1.5 mJ. In the simulations, the laser spot diameter was set to 40–50 μm, which provided a balance between spatial resolution, energy density control, and suppression of thermal effects, thereby enhancing the uniformity of the intensity distribution and the versatility of the process.

**Figure 4:**
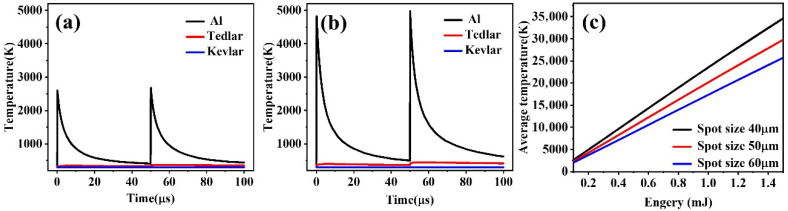
Temperature variation curves of Al for laser pulse energies of: (**a**) 0.1 mJ, (**b**) 0.2 mJ during a 100 μs interval. (**c**) Trends in the average temperature of the Al layer under different laser energies and spot sizes.

Figure 4a shows that at a lower pulse energy of 0.1 mJ, the Al layer absorbs a portion of the laser energy, converting it into thermal energy. The heat then transfers into the material through an unsteady thermal conduction mechanism. When the local temperature rises rapidly to its melting point (933 K), a solid-to-liquid phase transition occurs, indicating initial melting behavior. As the pulse energy increases to 0.2 mJ (Figure 4b), the energy absorbed at the surface of the Al layer accumulates rapidly, causing the temperature to surge above 2600 K, thereby initiating a continuous solid–liquid-gas multiphase transition process. This process is accompanied by intense latent heat release and material morphological reconstruction, with liquid metal rapidly evaporating under strong temperature gradients, resulting in high-density metal vapor formation. At this stage, auxiliary gas creates dynamic pressure disturbances in the laser irradiation area, effectively dispersing the vapor and etching out regions of removed material, leading to the formation of micron-sized etching pits with distinctive geometric profiles on the surface of the Al layer.

As the laser pulse energy further increases, the heat flow to deeper material layers is enhanced, significantly raising the temperature of the underlying Tedlar material. As shown in Figure 4c, when the pulse energy exceeds 0.2 mJ and the spot diameter remains within the range of 40–50 μm, the laser beam matches well in terms of energy density and spatial distribution, enabling stable vaporization and removal of the Al layer. This provides a fundamental basis for localized thermodynamic control and phase transition management in subsequent selective laser machining of the multilayer structure.

**Figure 5:**
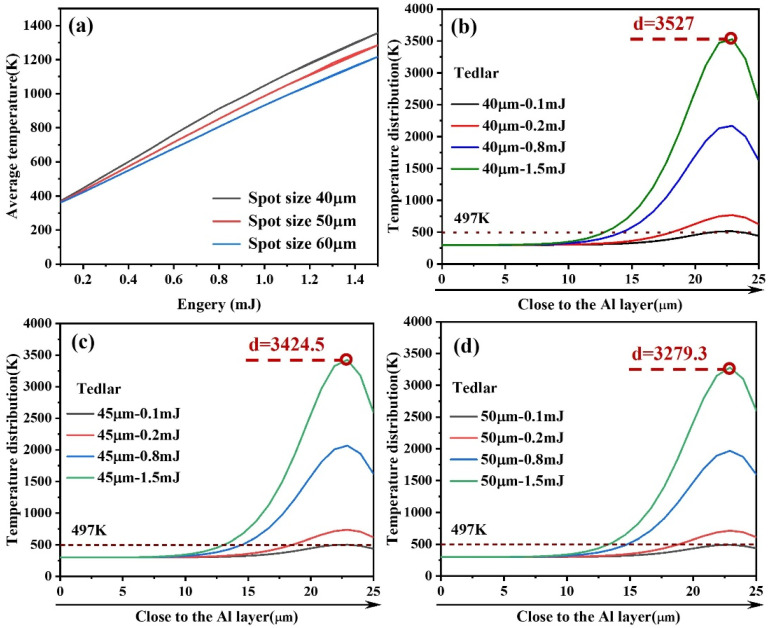
(**a**) Average temperature changes in Tedlar with variations in laser energy and spot size; Temperature distribution in Tedlar along the laser beam axis at (**b**) 40 μm, (**c**) 45 μm, and (**d**) 50 μm.

Building on the etching of the Al layer, the study further investigates the temperature evolution and underlying physical mechanisms of the Tedlar layer during laser irradiation. As illustrated in Figure 5a, as the laser pulse energy increases from 0.1 mJ to 1.5 mJ, the average temperature of the Tedlar layer exhibits an approximately linear rising trend, indicating a close correlation between temperature increase and thermal input. To analyze the heat transfer mechanisms in greater depth, a quantitative analysis of the temperature response under laser action was conducted using a thermal conduction model.

(5) $$\frac{\partial T}{\partial t}=\alpha \nabla^{2}T+\frac{Q_{input}}{\rho C_{p}}$$

In this context, T represents the temperature field, α is the thermal diffusivity, ρ denotes material density, C_p_ is the specific heat capacity, and Q_input_ is the input laser energy per unit volume. This equation reveals a positive correlation between laser thermal input and material temperature increase.

As a polymer material, Tedlar exhibits low thermal conductivity, which restricts heat diffusion within the layer and readily induces significant local temperature gradients, leading to thermal stress concentration and structural changes. When the pulse energy exceeds 0.25 mJ, the average temperature of the Tedlar layer surpasses its melting point (497 K), indicating the onset of melting. With further increases in laser energy up to 1.5 mJ, the melt depth in the Tedlar layer continues to grow, reaching approximately 12 μm. This reflects a typical deepening of the heat-affected zone, demonstrating that laser thermal input influences not only surface temperature rise but also substantially governs heat transfer and phase transition behavior within the material.

Under constant pulse energy conditions, the spot size significantly impacts the thermal response behavior of the material, as shown in Figure 5b–d. As the spot diameter increases, the energy density per unit area decreases markedly, leading to a reduced heat deposition rate in localized regions and consequently slowing the surface temperature rise. Furthermore, a larger irradiation area promotes radial heat diffusion, enhancing lateral thermal conduction effects, which effectively alleviates local temperature gradients and inhibits the formation of temperature peaks. From the thermodynamic mechanism perspective, this process reflects the synergistic effect between energy spatial distribution and material thermal diffusion capabilities; that is, a more uniform energy distribution results in a smoother local temperature rise and a more stable evolution of the thermal field.

**Figure 6:**
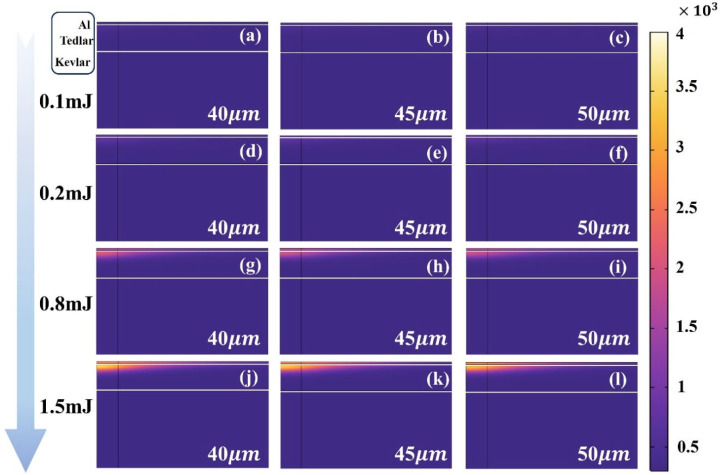
Temperature distribution in the three layers of material at laser pulse energies of (**a**–**c**) 0.1 mJ, (**d**–**f**) 0.2 mJ, (**g**–**i**) 0.8 mJ, and (**j**–**l**) 1.5 mJ for spot sizes of 40, 45, and 50 μm.

To elucidate the thermal response characteristics of the Al–Tedlar–Kevlar heterogeneous structure, Figure 6 presents the influence of laser pulse energy and spot size on the temperature distribution across the three layers. The results indicate that the Kevlar layer remains near room temperature with negligible thermal response, confirming that the thermal field is largely confined to the Al–Tedlar layers and that Kevlar decomposition is unlikely. Meanwhile, the Tedlar layer serves as a thermal shield, blocking direct laser interaction with the Kevlar substrate, but itself exhibits high sensitivity to laser input. Its temperature rises markedly, with heat distribution concentrated on the side adjacent to the Al layer, where heat transfer predominantly occurs along the interface, creating a steep temperature gradient. When the pulse energy reaches 0.5 mJ, the temperature within the Tedlar region approximately 8 μm below the Al interface exceeds the melting point, inducing local melting and initiating a thermally driven phase change. With further increases in pulse energy, both the depth of melting and the overall temperature rise significantly, reflecting the dominant role of heat accumulation and the intensification of the local thermal field. This behavior underscores the limited thermal diffusion in low-conductivity polymers, which promotes heat localization and the formation of temperature peaks. In addition, interfacial thermal resistance critically governs cross-layer heat transfer, strongly influencing the efficiency of thermal transport from the upper to the lower layers.

### 3.2. Impact of Laser Repetition Frequency on Al–Tedlar–Kevlar Composite Film

To investigate the regulatory effects of laser pulse repetition frequency on the thermal response behavior of the Al–Tedlar–Kevlar composite film, an analysis was conducted under conditions of a single pulse energy of 1mJ, pulse width of 120 ns, and a spot diameter of 45 μm. The temperature evolution and thermal diffusion characteristics were examined at frequencies of 10 kHz, 30 kHz, and 50 kHz.

**Figure 7:**
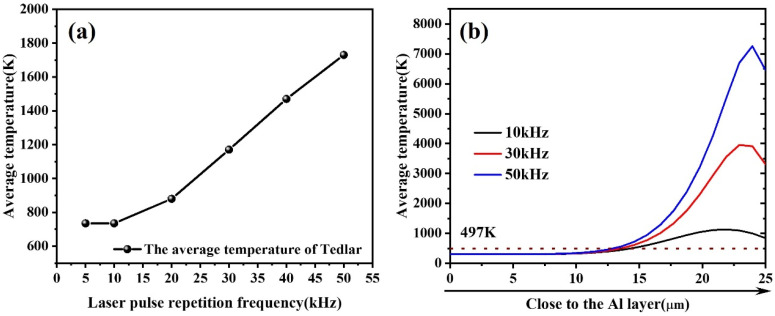
Temperature variation with changes in pulse repetition frequency: (**a**) Average temperature variation in Tedlar; (**b**) Temperature distribution in Tedlar along the beam axis.

The simulation results (Figure 7a) indicate that the average temperature of the Tedlar layer increases significantly with rising pulse frequency, showing an approximately linear growth after exceeding 15 kHz. This suggests that, under high-frequency conditions, there is an increase in the energy input per unit time, thereby enhancing the heat accumulation effect. However, as shown in Figure 7b, with the increase in repetition frequency, the thickness of the region within the Tedlar layer where the temperature exceeds the melting point (497 K) gradually decreases. This indicates that, while high frequencies raise the peak temperature, the effective depth of thermal diffusion is limited, resulting in a shallower melt zone.

**Figure 8:**
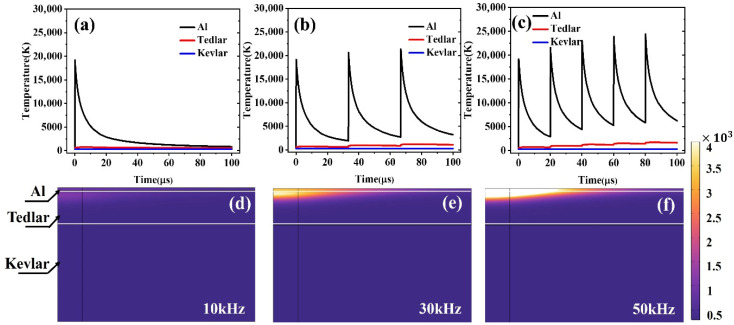
Temperature variation in the three materials within 0–100 μs as a function of laser pulse repetition frequency: (**a**) 10 kHz, (**b**) 30 kHz, (**c**) 50 kHz. Temperature distribution with changes in pulse repetition frequency: (**d**) 10 kHz, (**e**) 30 kHz, (**f**) 50 kHz.

Further comparison of the thermal responses of the component materials at different frequencies (Figure 8a–c) reveals that as the frequency increases, the local temperature rises significantly; however, the high-temperature region becomes concentrated toward the center of the spot, resulting in a reduction in the heat-affected zone (HAZ) extent, exhibiting greater thermal localization. This phenomenon can be attributed to the rapid accumulation of heat during high-frequency pulse inputs, while insufficient diffusion time restricts the heat to the irradiated core area.

Figure 8d–f illustrate the evolution of the average temperature of the Al–Tedlar–Kevlar structure within the 0–100 μs time range. The results show that at a low repetition frequency of 10 kHz, the pulse interval is much longer than the beam residence time on the same point along the scanning path, so each location is subjected to only a limited number of pulses, and thermal accumulation remains weak. In this case, the temperature curve exhibits a rapid rise followed by a sharp decay, indicating that after heat input, thermal energy diffuses promptly and the system is dominated by transient heat transfer. When the frequency increases to 30 kHz or higher, the pulse interval gradually approaches the thermal relaxation time of the material, enabling effective superposition of successive pulses at the same position. The temperature curve then displays a stepwise rise, where the temperature is lifted again before fully decaying in each cycle, leading to progressive accumulation of the thermal field and a continuous, stable heating trend. This thermal accumulation effect arises when the rate of energy input exceeds the material’s heat dissipation capacity, causing localized buildup of heat in both space and time, thereby amplifying the local temperature response. Such concentrated heating can, on the one hand, enhance local processing precision and reduce thermal interference in adjacent regions, but on the other hand, may also increase the risk of thermal damage.

### 3.3. Impact of Laser Pulse Width on Al–Tedlar–Kevlar Composite Film

**Figure 9:**
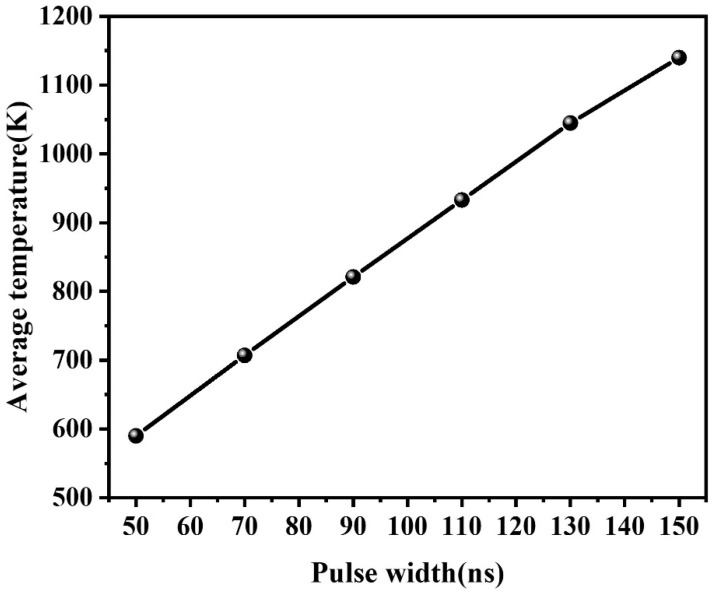
Temperature distribution in Tedlar with changes in pulse width from 50 to 150 ns.

**Figure 10:**
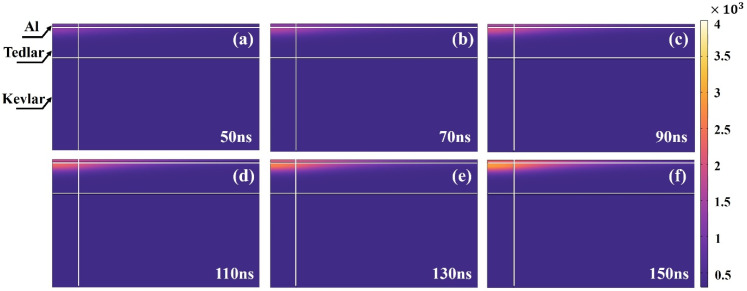
Temperature distribution in Tedlar with changes in pulse width from 50 to 150 ns: (**a**) 50 ns, (**b**) 70 ns, (**c**) 90 ns, (**d**) 110 ns, (**e**) 130 ns, (**f**) 150 ns.

At a pulse energy of 1 mJ (20 kHz, 45 μm spot), varying the pulse width (50–150 ns, in 10 ns increments) under constant energy conditions has almost no effect on the average temperature or spatial distribution within the Al–Tedlar–Kevlar composite film. This outcome aligns with physical expectations: since the total absorbed energy remains constant and the heat diffusion time is much longer than the nanosecond-scale pulse width, the temporal distribution of energy becomes negligible. In contrast, under constant peak power conditions (8.3 kW, Figure 9), increasing the pulse width raises the single-pulse energy, resulting in an approximately linear increase in average temperature and enhanced heat accumulation (Figure 10). In summary, pulse width plays a limited role under fixed energy, but becomes a key parameter for controlling thermal input and heat accumulation under fixed peak power.

### 3.4. Impact of Laser Parameters on Thermal Stress Behavior

To further investigate the potential effects of thermal–mechanical coupling during the laser processing of the Al–Tedlar–Kevlar composite film structure, the spatial distribution and peak response of stress within the film were analyzed under varying conditions of laser pulse energy, repetition frequency, and pulse width, with a particular focus on the evolution of thermal stress in the intermediate Tedlar layer due to uneven thermal expansion.

**Figure 11:**
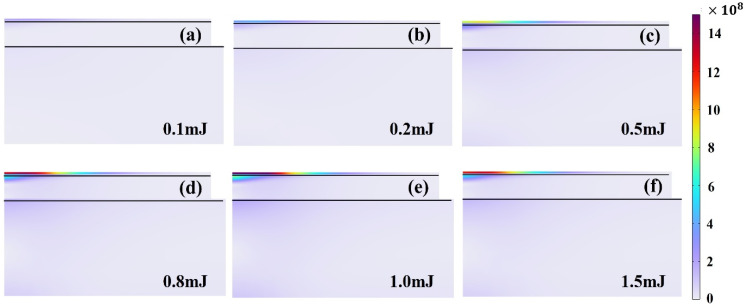
Stress distribution in the three-layer material with varying laser pulse energy at (**a**) 0.1 mJ, (**b**) 0.2 mJ, (**c**) 0.5 mJ, (**d**) 0.8 mJ, (**e**) 1.0 mJ, and (**f**) 1.5 mJ.

Figure 11 illustrates the distribution characteristics of equivalent stress within the Tedlar layer and the trend of its maximum stress values under different laser pulse energy conditions. The results show that under a laser pulse repetition frequency of 20 kHz, a spot diameter of 45 μm, and a pulse width of 120 ns, as the pulse energy increases from 0.1 mJ to 1.5 mJ, the temperature within the Tedlar layer rises significantly, leading to intensified thermal expansion mismatch and a consequent increase in internal stress levels. Given that the tensile modulus of Tedlar material is approximately 2.1–3.0 GPa, the simulation results show that when the laser energy exceeds 0.8 mJ, the peak stress values approach or even surpass 2.1 GPa, suggesting that the material may be nearing its elastic limit and could be at risk of undergoing plastic deformation or microcrack initiation. Therefore, under the influence of a high-energy pulsed laser, there is a critical need to closely monitor the potential for thermal damage and degradation of structural integrity in the Tedlar layer.

**Figure 12:**
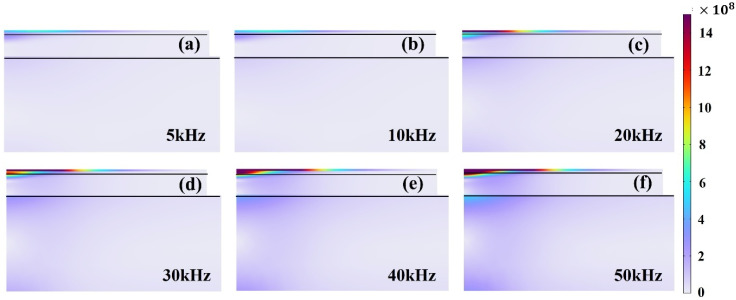
Variation in stress with changes in pulse repetition frequency: (**a**) 5 kHz, (**b**) 10 kHz, (**c**) 20 kHz, (**d**) 30 kHz, (**e**) 40 kHz, (**f**) 50 kHz.

By keeping the single-pulse energy (1 mJ), pulse duration (120 ns), and spot diameter (45 μm) constant, the laser repetition frequency was varied from 5 kHz to 50 kHz to investigate the thermal response of the Tedlar layer. As shown in Figure 12, increasing frequency leads to a pronounced rise in stress levels, particularly beyond 20 kHz, due to insufficient cooling time between successive pulses. The resulting progressive temperature buildup and steep thermal gradients generate significant stress concentrations, while repeated heating at the same location further exacerbates the risks of thermal fatigue and structural failure. These findings highlight the fundamental physical characteristics of short-duration multipulse irradiation—namely, enhanced thermal localization and heightened susceptibility to thermal fatigue and localized structural damage. Nevertheless, since the present simulations are confined to short timescales and single-scan conditions, prolonged irradiation or multiple scanning passes may induce cumulative effects such as global warping, interfacial delamination, or fatigue cracking, which warrant dedicated long-term multipulse investigations in future work.

**Figure 13:**
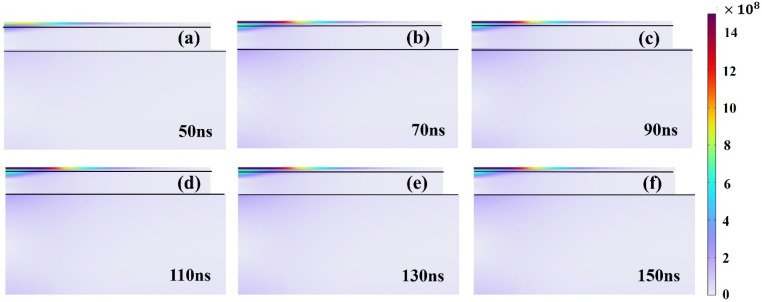
Stress distribution in Tedlar with changes in pulse width from 50 to 150 ns: (**a**) 50 ns, (**b**) 70 ns, (**c**) 90 ns, (**d**) 110 ns, (**e**) 130 ns, (**f**) 150 ns.

Figure 13 illustrates the evolution of stress under different pulse width conditions while maintaining a constant laser peak power. It is evident that as the pulse width increases, the duration of thermal input is extended, and the range of heat diffusion is enhanced, resulting in a more pronounced temperature gradient within the Tedlar layer, which consequently leads to an increase in thermal stress. Although longer pulses help to reduce the instantaneous temperature peaks, they accumulate greater heat, thereby exacerbating the non-uniform expansion of the material and elevating the overall levels of thermal stress.

Overall, the results indicate that laser parameters have a significant impact on the stress state of the Tedlar layer. Pulse energy directly governs the peak temperature and stress level, while pulse width and repetition frequency regulate heat diffusion and accumulation. Therefore, it is recommended to maintain the pulse energy below 0.8 mJ and adopt moderate frequencies and pulse widths to ensure that stress remains within the elastic limit. These results are obtained under the assumption of perfect interfacial bonding; however, actual Al–Tedlar–Kevlar composites inevitably contain defects that introduce thermal resistance and stress concentrations, potentially accelerating interfacial failure. Consequently, the reported results may represent an idealized lower bound. Despite these simplifications, our predicted interfacial delamination and polymer softening are consistent with experimental observations of laser-induced blistering, thermal damage, and fatigue-related structural failure in polymer–metal composites [28,29,30], which further supports the reliability of the proposed thermo–mechanical model.

### 3.5. Optimal Processing Parameters for Laser Etching

This study systematically examined the influence of key process parameters—pulse energy, repetition rate, spot diameter, and pulse duration—on the thermal response and interfacial stress evolution of Al–Tedlar–Kevlar composite films during laser micropatterning. The findings demonstrate that laser–material interaction is strongly governed by the temporal and spatial distribution of thermal input. A comprehensive assessment of thermal stability and stress regulation identified the optimal parameters as a pulse energy of 0.5 mJ, a repetition rate of 20 kHz, a spot diameter of 45 μm, and a pulse duration of 120 ns. Under these conditions, laser energy is precisely controlled, heat accumulation remains moderate, and thermal damage to the Tedlar and Kevlar layers is effectively prevented, yielding sharp pattern boundaries with a minimized heat-affected zone. Representative micrographs (Figures S1 and S2) further corroborate these results, showing morphological evolution from residual Al at low energies, to clean removal under optimal conditions, and to substrate damage at higher inputs—closely consistent with the simulated threshold behavior and providing strong support for the proposed model. The identified parameter trends provide practical guidance: higher pulse energy or lower laser pulse repetition frequency enhances throughput but increases thermal accumulation and defect risk. By contrast, optimizing peak power and pulse width under fixed energy stabilizes the thermal response, offering useful guidelines to balance efficiency and quality in real-world applications.

## 4. Conclusions

This study focuses on the Al–Tedlar–Kevlar composite film structure and, using a finite element multiphysics simulation approach, systematically investigates the combined effects of laser repetition frequency, pulse energy, and pulse width on the temperature distribution and thermal stress evolution during the laser etching process, with particular emphasis on elucidating the critical role of parameter control in ensuring structural stability and processing quality of the film layers. The main conclusions are as follows:

1. Effect of Repetition Frequency: The laser repetition frequency significantly influences heat accumulation and stress evolution. An increase in frequency raises the heat input density and reduces the thermal diffusion interval, resulting in a sharp rise in localized temperature. Particularly when the frequency exceeds 20 kHz, distinct thermal stress concentration zones form within the material, increasing the risk of membrane cracking and interfacial delamination. Therefore, a balance must be achieved between processing efficiency and thermal stability.
2. Effect of Pulse Energy: Pulse energy is a dominant factor determining the intensity of thermal excitation and the magnitude of stress. When the energy exceeds 0.8 mJ, the stress levels in the Tedlar layer approach its minimum tensile modulus (approximately 2.1 GPa), leading the material to likely enter the plastic deformation stage and potentially initiate micro-cracking. While higher energy can enhance etching efficiency, excessive energy input can severely compromise the structural integrity and service life of the membrane.
3. Effect of Pulse Width: Pulse width significantly influences the thermo-mechanical response of the Al–Tedlar–Kevlar composite film. At fixed pulse energy, its effect on the temperature field is minimal, with a stable overall response. Under constant peak power, however, longer pulses increase per-pulse energy and extend thermal coupling, intensifying heat accumulation, temperature gradients, nonuniform expansion, and thermal stresses. Thus, in high–peak power laser processing, pulse width governs both thermal input and stress evolution, making it a critical parameter for optimization.

In summary, the synergy between laser repetition frequency, pulse energy, and pulse width collaboratively determines the thermal–stress coupling behavior of the composite layers during laser etching. This study not only elucidates the intrinsic principles governing the thermal–mechanical responses to processing parameters but also provides a theoretical foundation and workable parameter window for optimizing precision laser processing techniques.

## Figures and Tables

**Table 1:** Physical Properties of the Three Materials.

Physical Parameters	Al	Tedlar	Kevlar
Specific heat (J/kg·K)	900~1050	1010	1400~2500
Density (kg/m^3^)	2700	1500	1440
Thermal conductivity (W/(m·K)	235	0.206	1.7
Real part of refractive index	1.3763	1.46	/
Imaginary part of refractive index	10.245	10^−6^	/

## Data Availability

The original contributions presented in this study are included in the article and Supplementary Materials. Further inquiries can be directed to the corresponding author.

## Supplementary Materials

The following supporting information can be downloaded at: https://www.mdpi.com/article/10.3390/ma18214839/s1, Figure S1: Representative surface micromorphologies of the composite under different laser pulse energies: (a) residual aluminum remaining at 0.2 mJ, (b) clean removal with intact substrate at 0.5 mJ (optimal condition), and (c) substrate damage evidenced by ablation pits at 0.8 mJ. The observed evolution is consistent with the threshold behavior predicted by the simulations; Figure S2: Representative surface micromorphologies of the composite under different laser repetition frequencies at a fixed pulse energy of 0.5 mJ: (a) residual aluminum remaining at 10 kHz, (b) clean removal with an intact substrate at 20 kHz (optimal condition), and (c) substrate damage evidenced by local ablation at 30 kHz. The observed evolution is consistent with the threshold behavior predicted by the simulations; Figure S3: Representative surface micromorphologies of the composite under different laser pulse energies: (a) 0.1 mJ, showing incomplete removal with residual aluminum; (b) 0.2 mJ, exhibiting more effective removal but with residual traces; (c) 0.5 mJ, achieving clean removal with an intact substrate (optimal condition); (d) 0.8 mJ, where local substrate damage becomes visible; and (e) 1.5 mJ, showing pronounced substrate damage.; Figure S4: Representative surface morphologies of the composite under different laser repetition frequencies: (a) 5 kHz and (b) 10 kHz, showing incomplete removal with residual traces; (c) 20 kHz, achieving clean removal with an intact substrate (optimal condition); (d) 30 kHz and (e) 40 kHz, exhibiting localized substrate damage (highlighted in red); and (f) 50 kHz, showing severe substrate damage accompanied by bubble formation.

## References

[1] Zhang F.D., Ren P.G., Guo Z.Z., Wang J., Chen Z.Y., Zong Z., Hu J., Jin Y.L., Ren F. (2022). Asymmetric multilayered MXene-AgNWs/cellulose nanofiber composite films with antibacterial properties for high-efficiency electromagnetic interference shielding. J. Mater. Sci. Technol..

[2] Zhu Z., Lu H., Zhao W.J., Tuerxunjiang A., Chang X.Q. (2023). Materials, performances and applications of electric heating films. Renew. Sustain. Energy Rev..

[3] Kwon D.J., Kwon I.J., Milam-Guerrero J., Yang S.B., Yeum J.H., Choi H.H. (2022). Aramid nanofiber-reinforced multilayer electromagnetic-interference (EMI) shielding composites with high interfacial durability. Mater. Des..

[4] Xia C.L., Yu J., Shi S.Q., Qiu Y., Cai L.P., Wu H.F., Ren H., Nie X., Zhang H.L. (2017). Natural fiber and aluminum sheet hybrid composites for high electromagnetic interference shielding performance. Compos. Part B-Eng..

[5] Xue L.L., Xiong S.S. (2024). Flexible metal foil/polymer sandwich composites for electromagnetic interference shielding with anti-wind-sand environment tolerance. J. Mater. Sci.-Mater. Electron..

[6] Ursache S., Cerbu C., Hadar A. (2024). Characteristics of Carbon and Kevlar Fibres, Their Composites and Structural Applications in Civil Engineering-A Review. Polymers.

[7] Shang K., Wu G., Liu X., Yang J., Wang R. (2021). Femtosecond Laser Etching of Aluminum Film on Tedlar Composite Surfaces. Chin. J. Lasers.

[8] Ehrhardt M., Lai S., Lorenz P., Zimmer K. (2020). Guiding of LIPSS formation by excimer laser irradiation of pre-patterned polymer films for tailored hierarchical structures. Appl. Surf. Sci..

[9] Mezera M., van Drongelen M., Römer G. (2018). Laser-Induced Periodic Surface Structures (LIPSS) on Polymers Processed with Picosecond Laser Pulses. J. Laser Micro Nanoeng..

[10] Ali B., Litvinyuk I.V., Rybachuk M. (2021). Femtosecond laser micromachining of diamond: Current research status, applications and challenges. Carbon.

[11] Putz B., Völker B., Semprimoschnig C., Cordill M.J. (2017). Influence of extreme thermal cycling on metal-polymer interfaces. Microelectron. Eng..

[12] Emanuel M., Mahyari P., Maniscalco M., May N., Choi H., Bliznakov T., Moore T., Anaei M.T.M., Phoulady A., Blagojevic A. Comprehensive Simulation of Laser Processing Systems: From Source to Surface Interaction. Proceedings of the 19th Conference on Laser Based Micro and Nanoprocessing.

[13] Zhang C.H., Wang F., Zhang C.S., Bao Y.L., Wang M.J., Liu D., Wu J., Su Z.M. (2024). Thermal-force coupling simulation of carbon fibre-reinforced poly-ether-ether-ketone thermoplastic composites during the laser-assisted automated tape placement process. Plast. Rubber Compos..

[14] Bian J., Chen F., Yang B., Zhang X., Chen J., Zhuang S., Zhang X., Song J. (2020). Laser-Induced Interfacial Spallation for Controllable and Versatile Delamination of Flexible Electronics. ACS Appl. Mater. Interfaces.

[15] Chakraborty S., Park H.-Y., Ahn S.I. (2022). Copper Laser Patterning on a Flexible Substrate Using a Cost-Effective 3D Printer. Sci. Rep..

[16] Li X., Tang R., Li D., Luo H., Wang T., Zhang J. (2024). Investigations of the Laser Ablation Mechanism of PMMA Microchannels Using Single-Pass and Multi-Pass Laser Scans. Polymers.

[17] Said-Bacar Z., Leroy Y., Antoni F., Slaoui A., Fogarassy E. (2011). Modeling of CW laser diode irradiation of amorphous silicon films. Appl. Surf. Sci..

[18] von der Heide C., Seuthe T., Epple M., Nolte S., Chichkov B.N. (2020). Methodology of Selective Metallic Thin Film Ablation from Susceptible Polymer Substrate Using Pulsed Femtosecond Laser. Appl. Phys. A.

[19] Kashihara K., Tomita T., Inoue M., Koshizaki N. (2021). Simulation of Photothermal Synthesis Processes in Metal–Organic Framework Films under Laser Irradiation. ACS Appl. Nano Mater..

[20] Varlamov P., Marx J., Elgueta Y.U., Melnikov I., Temnov V.V. (2024). Femtosecond Laser Ablation and Delamination of Functional Magnetic Multilayers at the Nanoscale. Nanomaterials.

[21] Cengel Y.A., Turner R.H., Smith R. (2001). Fundamentals of Thermal-Fluid Sciences.

[22] Wang H.-B. (1979). Introduction to Tedlar Poly(vinyl fluoride) Films.

[23] Chen H., Ginzburg V.V., Yang J., Yang Y., Liu W., Huang Y., Du L., Chen B. (2016). Thermal Conductivity of Polymer-Based Composites: Fundamentals and Applications. Prog. Polym. Sci..

[24] Joe D.J., Kim S., Park J.H., Park D.Y., Lee H.E., Im T.H., Choi I., Ruoff R.S., Lee K.J. (2017). Laser-Material Interactions for Flexible Applications. Adv. Mater..

[25] Self S.A. (1983). Focusing of spherical Gaussian beams. Appl. Opt..

[26] Vora H.D., Santhanakrishnan S., Harimkar S.P., Boetcher S.K.S., Dahotre N.B. (2013). One-dimensional multipulse laser machining of structural alumina: Evolution of surface topography. Int. J. Adv. Manuf. Technol..

[27] Kumar K.K., Samuel G.L., Shunmugam M.S. (2019). Theoretical and experimental investigations of ultra-short pulse laser interaction on Ti6Al4V alloy. J. Mater. Process. Technol..

[28] Varlamov P., Timofeev V., Andreev A. (2024). Femtosecond Laser Ablation and Delamination of Thin Films on Substrates: Mechanisms and Dynamics. Nanomaterials.

[29] Ravi-Kumar S. (2019). Laser Ablation of Polymers: A Review. Prog. Org. Coat..

[30] Nath P., Patel R.S. (2020). Laser Processing of Polymer-Based Composites: A Review. Polymers.

