# Supplementary material for: Finite Element Analysis of Thermal–Mechanical Coupling and Process Parameter Optimization in Laser Etching of Al–Tedlar–Kevlar Composite Films
Source: Materials (Basel). 2025 Oct 23;18(21):4839. doi: 10.3390/ma18214839 (PMC12608983; doi:10.3390/ma18214839)
Supplement: Supplementary file 1 [file materials-18-04839-s001.zip › materials-3911406-supplementary.pdf]

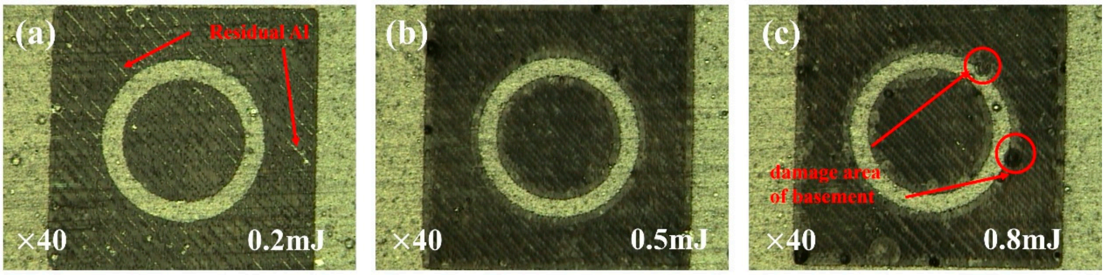

Figure S1. Representative surface micromorphologies of the composite under different laser pulse energies: (a) residual aluminum remaining at 0.2 mJ, (b) clean removal with intact substrate at 0.5 mJ (optimal condition), and (c) substrate damage evidenced by ablation pits at 0.8 mJ. The observed evolution is consistent with the threshold behavior predicted by the simulations.

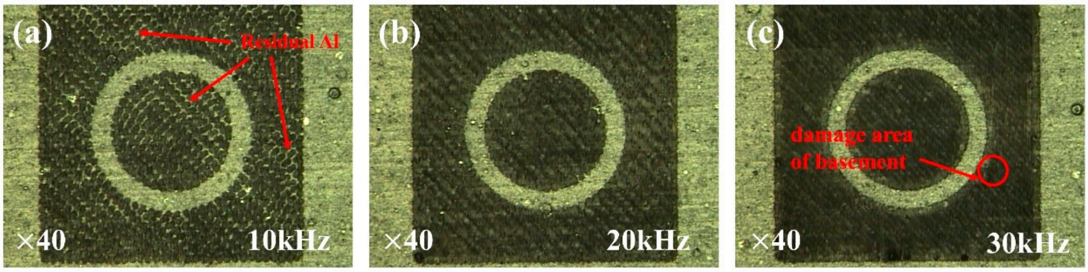

Figure S2. Representative surface micromorphologies of the composite under different laser repetition frequencies at a fixed pulse energy of 0.5 mJ: (a) residual aluminum remaining at 10 kHz, (b) clean removal with an intact substrate at 20 kHz (optimal condition), and (c) substrate damage evidenced by local ablation at 30 kHz. The observed evolution is consistent with the threshold behavior predicted by the simulations.

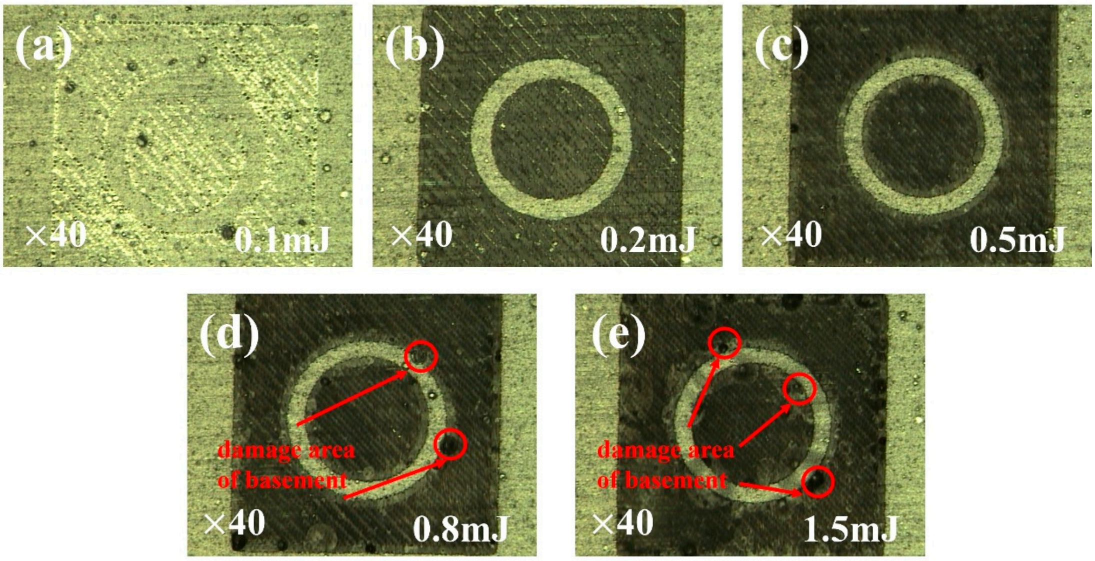

Figure S3. Representative surface micromorphologies of the composite under different laser pulse energies: (a) 0.1 mJ, showing incomplete removal with residual aluminum; (b) 0.2 mJ, exhibiting more effective removal but with residual traces; (c) 0.5 mJ, achieving clean removal with an intact substrate (optimal condition); (d) 0.8 mJ, where local substrate damage becomes visible; and (e) 1.5 mJ, showing pronounced substrate damage. The observed evolution is consistent with the threshold behavior predicted by the simulations and strongly supports the proposed model.

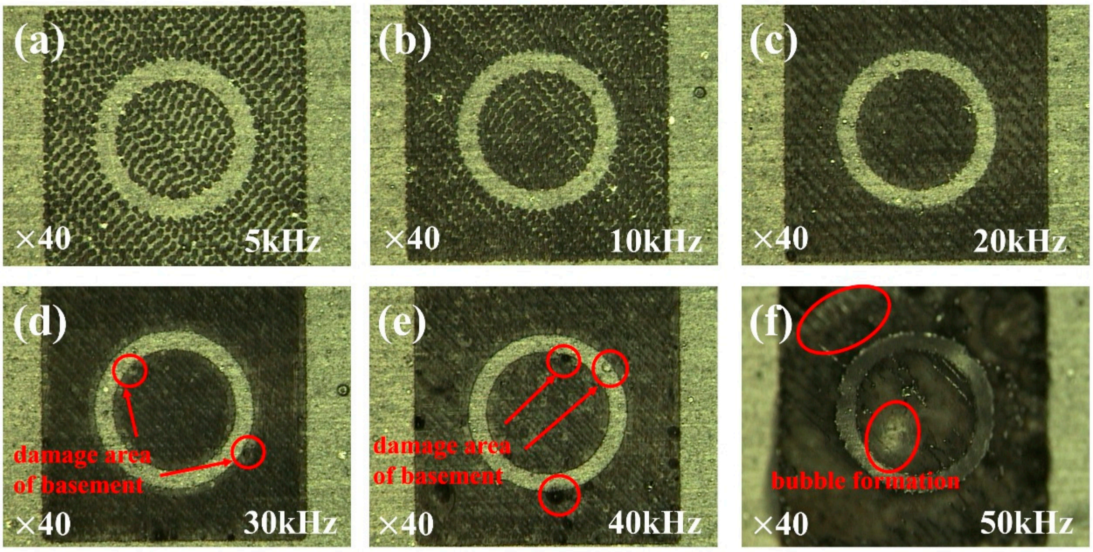

Figure S4. Representative surface morphologies of the composite under different laser repetition frequencies: (a) 5 kHz and (b) 10 kHz, showing incomplete removal with residual traces; (c) 20 kHz, achieving clean removal with an intact substrate (optimal condition); (d) 30 kHz and (e) 40 kHz, exhibiting localized substrate damage (highlighted in red); and (f) 50 kHz, showing severe substrate damage accompanied by bubble formation. The observed evolution reflects the threshold-dependent interaction between laser energy deposition and material response.
